# Changes in pain catastrophizing, fear-avoidance beliefs, and pain self-efficacy mediate changes in pain intensity on disability in the treatment of chronic low back pain

**DOI:** 10.1097/PR9.0000000000001092

**Published:** 2023-09-13

**Authors:** Truls Ryum, Tore C. Stiles

**Affiliations:** Department of Psychology, Norwegian University of Science and Technology, Trondheim, Norway

**Keywords:** Chronic low back pain, Fear-avoidance, Self-efficacy, Pain catastrophizing, Randomized controlled trial

## Abstract

**Introduction:**

Treatment of chronic low back pain (CLBP) based on the fear-avoidance model (FAM) has received support in randomized controlled trials, but few studies have examined treatment processes associated with treatment outcome. This study examined changes in pain catastrophizing, fear-avoidance beliefs, and pain self-efficacy as mediators of the relation between changes in pain intensity and disability in exposure-based treatment of CLBP.

**Methods:**

Data from a randomized controlled trial with 2 treatment arms (exposure treatment based on the FAM with/without in-session exposure) was pooled, including only participants with complete data (N = 69). Change scores (pre to booster session) were computed for all variables, and the indirect effect of change in pain intensity on change in 3 measures of disability, through change in the proposed mediators, was tested in parallel mediation analyses.

**Results:**

Decreases in pain catastrophizing and fear-avoidance beliefs, as well as increases in pain self-efficacy, mediated a unique proportion of the relation between changes in pain intensity and disability, depending on the outcome measure. The direct relation between changes in pain intensity and disability was absent when indirect effects were controlled.

**Conclusions:**

The results suggest that the way pain is interpreted (pain catastrophizing, fear-avoidance beliefs), as well as pain self-efficacy, are all more critical for reducing disability in exposure-based treatment of CLBP than symptom relief per se.

## 1. Introduction

Treatment of chronic low back pain (CLBP) based on the fear-avoidance model (FAM)^48,49^ has been supported in randomized controlled trials (RCTs)^16,22,37,56^ and case studies.^4,13,25,47,50^ Following an episode of acute pain, the FAM posits that it is not pain itself, but rather, *the interpretation* of pain that is essential for either a rapid recovery or the development of chronic pain.^23^ Pain that is perceived as nonthreatening is likely not to interfere substantially with daily activities, and functional recovery is rapidly promoted. However, pain that is misinterpreted as a sign of danger triggers a negative cascade of pain-related fear, catastrophizing, and avoidance behaviors, which may ultimately lead to disability.^23,48^ Thus, pain cognitions (eg, fear-avoidance, catastrophizing) are proposed as central to both the development and maintenance of pain-related disability, and patients' fear of pain and safety behaviors are challenged in treatment to improve functioning through graded, in vivo exposure to movements and tasks avoided due to fear of pain and/or (re)injury.^48^

Although outcome studies examine the overall effect of a treatment, process research may help clarify the underlying mechanisms associated with beneficial outcomes; that is, the intermediate variables or “mediators” associated with positive change. Surprisingly, only a handful of RCTs, comparing an active psychological intervention with that of a control condition, have examined mediators of treatment outcome in the context of CLBP,^8,29,40,41^ with only 2 based specifically on the FAM.^22,39^ However, results indicate that changes in key components of the FAM, including pain catastrophizing^8,22,40,41^ and fear of movement,^29,39^ are important for reducing disability. Support has also been found for the mediating role of pain self-efficacy,^8,39^ a concept stemming from social learning theories,^2^ which postulates that the degree of confidence a person has in conducting normal activities and tasks, despite experiencing pain, is essential for reducing disability with CLBP.^36^ The latter finding is important because the FAM is rooted within a learning theoretical framework, but the role of pain self-efficacy is often not highlighted, although the model has been revised since its inception.^12,55^

Findings from RCTs without a control condition may add to our understanding of treatment processes facilitating change with CLBP, although there are limitations with drawing causal inferences. Of particular interest is the interrelations between changes in pain intensity and disability, which are recommended as outcomes in clinical trials with CLBP,^46^ and intermediate variables (“mediators”) posited to facilitate change. Although the importance of reducing pain for improving functioning has sometimes been downplayed,^19,43^ and the FAM emphasizes change in pain-related cognitions as primary for improvement, we are not aware of research examining if there is a (1) direct relation between changes in pain intensity and disability or, rather, if this effect is (2) mediated through changes in intermediate mediators (fear-avoidance beliefs, pain catastrophizing, and pain self-efficacy). Alternatively, changes in pain and disability could be unrelated (3).

Utilizing data from an RCT examining the effect of an exposure-based treatment for CLBP,^37^ the aim of this study was thus to examine if changes in pain catastrophizing, fear-avoidance beliefs, and pain self-efficacy mediate changes in pain intensity on 3 measures of disability. We wanted to examine the unique contribution of these mediators simultaneously, in parallel mediation analyses, while also acknowledging that disability may be operationalized in distinctive ways (ie, globally vs behavior-specific measures of physical functioning). We hypothesized that all proposed mediators would demonstrate indirect effects but made no specific prediction as to their unique contribution.

## 2. Method

### 2.1. Study design

This study utilizes pre-session and booster-session data from a 2-armed, randomized, controlled trial^37^ (“Dare to move,” Clinical Trials NCT01158339), which examined the effect of in-session exposure (ISE) of feared movements in fear-avoidance (FA) treatment of CLBP. Participants in both treatment conditions received a group-based intervention based on the FAM,^1^ which followed a similar outline. The use of ISE was the only unique feature differentiating the 2 treatment conditions (FA-ISE vs FA). Both treatments consisted of 5 weekly sessions (1.5 hours), followed by a booster session at 7-weeks posttreatment. Participants in both conditions demonstrated significant improvements on primary and most secondary outcomes, with treatment effects upheld at 1-year follow-up, and mostly nonsignificant between-group differences.^37^Full details on design, participants, procedures, and outcomes are discussed below.

### 2.2. Participants

Inclusion criteria in the original trial were unspecified CLBP for more than 3 months localized from L1 to S1, severe pain that reduced working capacity and quality of life (on sick leave), pain caused not by nerve root affection, adequate fluency in Norwegian to be able to participate in group activities, regular work to return to, and between 18 and 60 years of age. Participants did not have to meet a prespecified level of fear-avoidance beliefs to be eligible. Exclusion criteria were 100% permanent disability, CLBP secondary to other somatic or psychiatric disorders, alcohol and drug abuse, CLBP caused by ankylosing spondylitis and other spondylarthopaties, indication for back/spine surgery or performed back surgery during the past 12 months, “red flags” (eg, bladder- and anal paresis), ongoing insurance affairs for all types of sickness, injuries, and accidents, and use of medication known to cause psychiatric symptoms.

Ninety patients were randomized to 1 of the 2 treatment conditions, but outcome data from 9 patients were missing. As this study examined variables during the active treatment phase, we only included participants with complete pre-session and booster-session data. The total sample in this study was thus N = 69 (FA-ISE: n = 37; FA: n = 32), except for the analyses using the back performance scale (BPS) (n = 61 due to missing data). This sample is identical to the completer sample in the original trial.^37^

### 2.3. Randomization

Patients who met inclusion criteria and gave written consent to participate in the trial were randomized to FA-ISE or FA. Allocation ratio was 1:1. Randomization was not stratified according to any specific target variable(s). The original clinical trial was approved by the Regional Committee for Ethics in Medical Research in Norway and conducted in accordance with the Declaration of Helsinki.

### 2.4. Interventions and therapists

Participants in both treatment conditions received an exposure-based intervention for CLBP, based on the FAM,^51^ which followed a similar outline. The treatment manuals consisted of a mixture of (1) psychoeducation, (2) cognitive restructuring, and (3) homework assignments. Participants were introduced to a cognitive–behavioral perspective on the vicious circle of fear-avoidant behaviors and its consequences and reassured that they would gradually be able to perform feared movements in a normal way, without the use of safety behaviors, restore functioning, and resume daily activities. Safety behaviors were identified and challenged, according to an individualized hierarchy of exposure tasks for each participant, and homework assignments were reviewed at the beginning of each session to help generalize treatment effects.

Both group treatments (FA; FA-ISE) were led by 2 therapists, a medical doctor and a physiotherapist. The medical doctor was a specialist in physical medicine and rehabilitation as well as rheumatology, a qualified specialist in cognitive therapy with extensive experience with patients with chronic pain, and specifically trained in the application of the FAM. In addition, 3 experienced physiotherapists were recruited as cotherapists. All therapists received specific training from one of the authors (T.C.S.) in the use of the manual before the study and had to achieve an acceptable level of adherence and competence to be included as a therapist. All participating therapists received systematic supervision every 4 weeks during the trial, based on the feedback of videotaped treatment sessions.

Checks of treatment differentiability demonstrated that the use of ISE was pronounced in the FA-ISE condition but almost absent in the FA condition. Treatment conditions did not differ for therapist adherence and competence in reviewing homework assignments or in the amount of between-session exposure of feared movements.^37^

### 2.5. Measures

We included one measure of pain intensity, 3 measures of disability, and measures of the 3 proposed mediators. All variables were measured at pretreatment and after a booster-session 7 weeks after treatment.

#### 2.5.1. Pain intensity

*Average pain intensity* (API) during the past 24 hours was measured with one item from the Brief Pain Inventory (BPI),^10^ rated on a scale from 0 (no pain) to 10 (pain as bad as you can imagine). The BPI has demonstrated adequate internal consistency and construct validity with CLBP^20^ and was included as primary outcome in our original trial.

#### 2.5.2. Measures of disability

We included 3 measures of disability with distinct qualities. The Work and Social Adjustment Scale (WSAS) and the BPS were used as primary outcomes in the original trial.^37^ A third measure, the Physical Functioning Scale (PFS), was used as a secondary outcome in the original trial.

The *Work and Social Adjustment Scale* (WSAS)^30^ is a 5-item measure of functional impairment attributable to an identified problem according to 5 domains (work, home, social, private leisure, and interpersonal relations), rated on a scale from 0 (no impairment at all) to 8 (very severe impairment). The WSAS is a reliable and valid measure of impaired functioning,^34^ with excellent internal consistency, convergent/divergent validity, and test–retest reliability across various disorders.^9,31^ Higher scores reflect more severely impaired functioning.

*Physical function* was measured with the physical functioning scale (PFS) from the Short Form Health Survey (SF-36).^53^ The PFS consists of 10 items assessing limitations in common physical activities due to health problems, such as bathing, dressing, climbing stairs, and bending. Each item is rated on a 3-point scale from 3 (No, not limited at all) to 1 (Yes, limited a lot). This study utilized a 0 to 2 scale and reversed scoring, with a higher total score representing more limitations in performing physical activities. The SF-36 has been found to have adequate internal consistency, test–retest reliability, and construct validity with various patient populations.^7,32,33^ In comparison to the WSAS, the PFS provides a more behavior-specific measure of physical disability.

*Physical performance* was measured with the BPS,^42^ which is an observer-based performance test. The BPS combines 5 physical tests (sock test, pick-up test, roll-up test, fingertip-to-floor test, and lift test), all requiring sagittal plane mobility, rated on a scale from 0 (good performance) to 3 (substantially limited performance). The BPS has been found to be a reliable (intertester and test–retest) and valid measure of activity limitation.^28,42^ An independent assessor, blind to treatment condition, was responsible for these assessments.

#### 2.5.3. Mediators

All proposed mediators were included as secondary outcomes in the original trial.^37^

*Pain catastrophizing* was assessed with the pain catastrophizing scale (PCS),^45^ consisting of 13 items. Patients are asked to what extent they experience certain thoughts or feelings during pain on a 5-point rating scale from 0 (not at all) to 4 (always) (eg, “I worry all the time about whether the pain will end”; “I become afraid that the pain will get worse”), with higher scores reflecting higher levels of pain catastrophizing. Good internal consistency and test–retest reliability have been reported for the PCS total score with various pain samples,^54^ as well as acceptable construct validity in patients with low back pain.^14^

*The Fear and Avoidance Beliefs Questionnaire* (FABQ)^52^ is a 16-items measure assessing beliefs concerning how work (11 items) and physical activity (5 items) affect low back pain. Only the 11 items loading on the 2 suggested subscales were used in this study in an aggregated score (eg, “Physical activity may harm my back”; “I should not do my normal work with my present pain”). Items are rated from 0 (completely disagree) to 6 (completely agree). Adequate test–rest reliability, as well as construct and criterion validity, has been reported for patients with CLBP.^15^

*Pain self-efficacy* was measured with 3 items from the Arthritis Self-Efficacy Scale (ASES),^27^ one from each of the 3 main factors (pain management, physical function, and other symptoms) (eg, “How certain are you that you can continue most of your daily activities?”). Each item is rated on a scale from 0 (very uncertain) to 100 (very certain), with higher scores reflecting stronger pain self-efficacy. Adequate internal consistency reliability, as well as test–retest reliability and construct validity, for the full scale has been reported.^6^ Cronbach alpha for the 3-item scale in this study was good (α = 0.83 pretreatment and α = 0.86 posttreatment).

### 2.6. Statistical analyses

**Figure 1.**
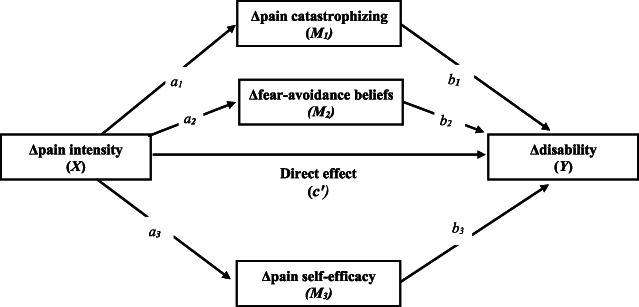
Effect pathways. The magnitude of the indirect effect for each mediator is calculated by multiplication of the indirect pathways (eg, *a*_*1*_ *b_*1*_ for Δpain catastrophizing). The total effect (C) equals the sum of the direct effect and the indirect effect(s) in each particular mediation analysis.

Data were analyzed using IBM SPSS Statistics 28. Pearson product moment correlation was used to examine the relationship between the variables of interest (pre to booster change scores [∆]), and mediation models were tested using the PROCESS macro^17^ (version 3.5; model 4), as depicted in Figure 1. A confidence interval of 95% and 10,000 bootstrap samples were used. Associations are mainly reported in unstandardized β coefficients.

Change scores (∆) were computed by subtracting pretreatment scores from the booster-session scores. Three parallel mediation analyses were computed, one for each disability measure, with each putative mediator (ΔPCS (M_1_)); (ΔFABQ (M_2_)); (∆ASES (M_3_)) tested simultaneously while accounting for the shared variance between them. Mediators are allowed to correlate but not causally influence each other, allowing for an estimation of the unique contribution of each mediator.

## 3. Results

### 3.1. Preliminary analyses

Independent samples *t*-tests (pretreatment) did not demonstrate statistically significant differences on any measure between participants with complete presession and booster session data, and participants with missing data (*P*s 0.08–0.91). Furthermore, independent samples *t*-tests did not demonstrate any statistically significant differences between treatment conditions (FA, FA-ISE) for the variables of interest at pretreatment- (*P*s 0.27–0.99) or posttreatment (*P*s 0.09–0.36).

### 3.2. Demographics and Pearson partial correlations among the study variables

Table 1 presents demographics, raw scores, change scores (∆), and a correlation matrix for the variables examined. At pretreatment, gender correlated significantly with FABQ (*r* = 0.26) and BPS (*r* = 0.26; men reporting higher scores), and age correlated significantly with BPS (*r* = 0.34; older participants scoring higher). Gender, age, and treatment condition were thus entered as covariates in the partial correlations and parallel mediation analyses to control for potential confounding effects. The use of pain medication was overall moderate pretreatment. Specifically, the use of strong opioids was absent, weak opioids (eg, Tramadol, Paralgin Forte) was limited (FA-ISE = 7; FA = 5), and both non-steroidal anti-inflammatory drugs (FA-ISE = 15; FA = 8) and paracetamol (FA-ISE = 19; FA = 19) was moderate. Use of benzodiazepines was absent. Chi-square tests did not demonstrate any significant between-group differences in the use of medication (*P*s < 0.17). For full information on demographics, please refer to our previous report.^37^

**Table 1:** Demographics, raw and change scores (Δ) with tests of between-treatment differences (fear-avoidance in-session exposure vs fear-avoidance) and partial correlations between change scores in the pooled patient sample.

	Pre Treatment					Booster Session				
	FA (n = 32)		FA-ISE (n = 37)		*P*	FA (n = 32)		FA-ISE (n = 37)		*P*
Demographics	N	%	N	%		N	%	N	%	
Gender (male)	9	28%	17	46%	0.13					
	N	SD	N	SD		M	SD	M	SD	
Age (years)	43.3	9.9	43.9	9.7	0.83					
Variables*										
API	3.9	1.7	3.8	1.6	0.88	2.8	1.8	3.7	2.2	0.09
WSAS	10.9	7.1	13.1	9.0	0.27	7.8	8.3	10.3	9.5	0.26
PFS	6.7	3.2	7.1	4.8	0.70	4.7	3.0	6.1	5.0	0.18
BPS	5.2	3.7	4.7	3.7	0.45	2.6	3.1	4.1	3.5	0.09
FABQ	28.4	14.8	29.3	16.6	0.82	18.6	15.8	25.2	17.9	0.12
ASES	52.2	25.9	52.1	30.6	0.99	71.7	23.6	64.1	27.6	0.23
PCS	15.5	10.3	14.3	8.7	0.58	9.9	9.2	12.1	10.1	0.36

The partial correlations supported the presence and direction of proposed relationships mostly in the moderate range (0.3 ≤ *r* < 0.5), according to Cohen.^11^ Importantly, the mediators (ΔPCS, ΔFABQ, ∆ASES) correlated only moderately in expected directions, implying that they measure distinct phenomena and not a common underlying construct. The correlation between ∆API and ∆BPS was not significant, but a statistically significant direct effect is not a necessary assumption to be met to test for indirect effects.^18^ Independent samples t-tests did not demonstrate any statistically significant differences between treatment conditions (FA vs FA-ISE) on the change scores (∆), except for the ∆BPS where the FA-condition outperformed the FA-ISE condition, as reported in the original clinical trial.^37^

### 3.3. Multiple mediation analyses

Results from the multiple mediation analyses are presented in Table 2. For ∆WSAS, only the confidence interval for the indirect effect of ΔPCS did not cross zero, indicating that the effect of ∆API on ∆WSAS was uniquely mediated through ∆PCS (standardized indirect effect = 0.12). Both ∆FABQ and ∆ASES emerged as significant and unique mediators of the effect of ∆API on ∆PFS (standardized effects: ∆FABQ = 0.11; ∆ASES = 0.13), with a total unstandardized indirect effect (both mediators combined) of 0.45 (ES = 72%) of the total effect, using the ratio of indirect effect to the total effect as a measure of effect size.^17^ Only ∆ASES mediated the effect of ∆API on ∆BPS (standardized indirect effect = 0.15), with an ES of 0.74%.

**Table 2:** Summary of separate parallel mediation analyses for each disability measure with change in pain catastrophizing scale, fear and avoidance beliefs questionnaire, and arthritis self-efficacy scale mediating the effects of change in pain intensity on change in functional disability.

IV	Total effect (path c)	Direct effect (path c')	Indirect effect*	ES	St. indirect effect	DV
Model 1						
ΔPCS (M_1_)	1.11 (0.39, 1.83)†	0.35 (−0.39, 1.10)	0.37 (0.07, 0.82)†	33%	0.12	
ΔFABQ (M_2_)	1.11 (0.39, 1.83)†	0.35 (−0.39, 1.10)	0.13 (−0.17, 0.45)	12%	0.04	
ΔASES (M_3_)	1.11 (0.39, 1.83)†	0.35 (−0.39, 1.10)	0.26 (−0.08, 0.66)	24%	0.09	ΔWSAS (n = 69)
Model 2						
ΔPCS (M_1_)	0.63 (0.18, 1.09)†	0.18 (−0.29, 0.67)	−0.02 (−0.19, 0.18)	3%	−0.01	
ΔFABQ (M_2_)	0.63 (0.18, 1.09)†	0.18 (−0.29, 0.67)	0.20 (0.01, 0.47)†	32%	0.11	
ΔASES (M_3_)	0.63 (0.18, 1.09)†	0.18 (−0.29, 0.67)	0.25 (0.04, 0.55)†	40%	0.13	ΔPFS (n = 69)
Model 3						
ΔPCS (M_1_)	0.23 (−0.05, 0.52)	−0.04 (−0.35, 0.28)	0.00 (−0.08, 0.08)	0%	0.00	
ΔFABQ (M_2_)	0.23 (−0.05, 0.52)	−0.04 (−0.35, 0.28)	0.10 (−0.02, 0.22)	43%	0.08	
ΔASES (M_3_)	0.23 (−0.05, 0.52)	−0.04 (−0.35, 0.28)	0.17 (0.01, 0.36)†	74%	0.15	ΔBPS (n = 61)

* The indirect effect quantifies how much 2 cases that differ by one unit on pain intensity (∆API) are estimated to differ on disability (∆WSAS, ∆PFS, and ∆BPS) through each putative mediator; ES = ratio of the indirect effect to the total effect as a measure of effect size.

† 95% confidence interval that does not include 0.

ASES, Arthritis Self-Efficacy Scale; BPS, back performance scale; DV, dependent variable; FABQ, fear-avoidance beliefs questionnaire; IV, independent variable; M_x_, mediator(s); PCS, pain catastrophizing scale; PFS, physical functioning scale; WSAS, Work and Social Adjustment Scale.

## 4. Discussion

Utilizing a sample of CLBP patients undergoing exposure-based treatment with/without ISE, the results from this study demonstrate that decreases in pain catastrophizing and fear-avoidance beliefs, as well as increases in pain self-efficacy, mediate a unique proportion of the relation between changes in pain intensity and functional disability, depending on how disability is operationalized. These findings are in accordance with previous clinical trials with experimental designs demonstrating cognitive factors (eg, pain-related fear, pain self-efficacy) to mediate outcome with CLBP,^8,22,29,39–41^ as also reported in a recent meta-analysis with chronic pain conditions more broadly.^35^ Our results add to the literature by demonstrating multiple pathways between changes in pain and disability and by showing that results may differ depending on how disability is assessed (ie, globally or behavior specific; self-rated or observer rated). The direct effect of change in pain intensity on disability was absent when indirect effects were controlled (or not present as for the BPS), indicating that cognitive variables, rather than symptom relief, improve functioning. All significant indirect effects explained a considerable proportion of the total effect for each disability measure (ES range 32%–74%).

Given that the intervention in both treatment conditions was based on the FAM, the finding that changes in pain catastrophizing and fear-avoidance beliefs mediate changes in pain intensity on disability is not surprising and in accordance with previous research.^22,39^ However, change in pain catastrophizing was only associated with improvement in general disability (WSAS) and not more behavior-specific measures of physical disability (PFS, BPS). Moreover, neither changes in fear-avoidance beliefs nor pain self-efficacy were unique mediators of general disability. Although it may be important to challenge catastrophic beliefs about pain for patients to develop more realistic attitudes towards daily activities in general, the results suggest that such changes are not likely to generalize to improvement in physical functioning specifically. Indeed, the FAM suggest that the tendency to catastrophize the experience of pain may primarily be a gateway into a vicious cycle of fear-related and avoidance-related behaviors, which may later become a chronic pain condition.^51^ It has also been suggested that catastrophizing may not only relate to the appraisal of pain but also serve an interpersonal purpose to elicit attention or support,^38,44^ but this was not examined in this study.

Increases in pain self-efficacy remained significant for improvement on 2 measures of physical disability (PFS and BPS), whereas an indirect effect for changes in fear-avoidance beliefs was only observed on the PFS. These results imply that reducing fear-avoidance beliefs, as well as enhancing pain self-efficacy, may be particularly important for improving physical functioning with CLBP and mediate the effect of change in pain intensity on disability. Fear-avoidance beliefs may be conceptualized as expectations about the consequences of certain actions, in relation to physical activity and/or work, and may be more proximal determinants of performing (feared) movements, compared with pain catastrophizing. Patients with higher levels of fear-avoidance beliefs tend to avoid activities to avoid provoking or aggravating pain,^26^ which may severely restrict normal function. The finding that pain self-efficacy was a stronger mediator compared with both fear-avoidance beliefs and pain catastrophizing was nonetheless surprising, given that the latter 2 are emphasized within the FAM.

The relative importance of fear-avoidance beliefs and pain self-efficacy in relation to the development and treatment of CLBP has been debated,^21^ and our results suggest that they may represent 2 parallel pathways for improving functioning with CLBP. Importantly, pain self-efficacy is not the mere absence of fear-avoidance beliefs because they both mediated a unique proportion of the relation between changes in pain intensity and disability (PFS). One may be fearful of a particular behavior one expects will aggravate pain, but the extent to which this behavior is performed or not will also depend on one's level of confidence in executing the behavior, despite pain.^36^ It has also been argued that there may be a conceptual overlap between measures of pain self-efficacy and disability, artificially inflating the correlation between the two, but the correlation was in the moderate range for fear-avoidance beliefs and disability, making this an unlikely cause for concern. Moreover, there is accumulating evidence from experimental RCTs demonstrating positive treatment outcomes with CLBP to be mediated through mechanisms common to all treatment models,^8,39^ as also reported with chronic pain conditions more broadly.^35^

Unlike most previous clinical trials examining the FAM with CLBP, the sample in this study did not consist of only highly fearful-avoidant patients. This may have attenuated the effects of the proposed mediators in the FAM, but cutoffs to identify high levels of catastrophizing are vague.^24^ Therefore, the results from this study may generalize well to the larger population of CLBP patients, and pain self-efficacy has also been found to mediate outcome in clinical trials with patients who catastrophize at high levels.^39^

Our results suggest that the way pain is interpreted, as well as pain self-efficacy, is more important for reducing disability in the treatment of CLBP than symptom relief.^3^ These results are line with a recent meta-analysis of mediation studies on back and neck pain, which reported significant mediating effects of self-efficacy, psychological distress, and fear between pain and the *development* of disability.^21^ Cognitive variables may thus play a key role both in the development and treatment of CLBP. Bearing in mind that this study did not include a control condition and may not determine if the proposed mediators need to be targeted to facilitate change, the results suggest that they should be monitored as indicators of treatment progress. There may be a risk in focusing exclusively on changing fear-avoidance beliefs, at the expense of cultivating coping and mastery experiences, which may also play a prominent role for treatment outcome, according to social learning theory.^2,5^ We thus advocate that treatment for CLBP should aim to challenge patients' catastrophic beliefs about pain and fear-avoidance beliefs, as well as increase their sense of pain mastery through creating realistic expectations, addressing functional concerns and solving problems in everyday life activities. The mediating effect of pain self-efficacy should probably be interpreted positively, but a caveat is that patients' level (and increase) in pain self-efficacy may depend on the use of avoidance and safety-behaviors when performing feared movements. The particularly strong effect of pain self-efficacy on the performance test (BPS) casts further suspicion on this assumption, which needs to be examined in future research.

This study has several strengths. First, we utilized data from a randomized controlled trial, with a longitudinal design and repeated measures, although without a control condition. Second, this is the first study examining the unique mediating role of 3 putative mediators between changes in pain intensity and 3 measures of disability in parallel mediation analyses. Some limitations also need to be acknowledged. First, caution is warranted in discussing cause and effect because all variables were measured at the same assessment points, which limit definitive conclusion regarding temporal relationships. Second, the sample-size was modest, increasing the risk of type II errors, and caution is warranted when interpreting the relative contribution of each mediator. Third, other mediators not examined in this study may have contributed to outcome, although psychiatric symptoms, for example, were low in this trial. Finally, we did not include a measure of race/ethnicity, which may have influenced on the generalizability of the findings.

In concluding, the results highlight the need to examine multiple mediators of treatment outcome simultaneously in clinical trials of CLBP. Evidence for pain catastrophizing, fear-avoidance beliefs, and pain self-efficacy mediating between changes in pain and disability were found. The results are relevant to the treatment of CLBP specifically but may extend to the management of chronic pain conditions more broadly.

## Disclosures

The authors have no conflict of interest to declare.
